# The evolution of bitter taste receptor gene in primates: Gene duplication and selection

**DOI:** 10.1002/ece3.10610

**Published:** 2023-10-13

**Authors:** Ping Feng, Xinyue Liang, Hongling Yu, Xiaoyan Dong, Qiufang Liang, Chuanyin Dai

**Affiliations:** ^1^ Key Laboratory of Ecology of Rare and Endangered Species and Environmental Protection, Ministry of Education of the People's Republic of China Guangxi Normal University Guilin Guangxi China; ^2^ Guangxi Key Laboratory of Rare and Endangered Animal Ecology Guangxi Normal University Guilin Guangxi China

**Keywords:** bitter taste receptor gene, Cercopithecidae species, primates, selective pressure

## Abstract

Bitter taste perception plays an important role in preventing animals from digesting poisonous and harmful substances. In primates, especially the Cercopithecidae species, most species feed on plants; thus, it is reasonable to speculate that most of the bitter taste receptor genes (*T2R*s) of primates are under purifying selection to maintain the functional stability of bitter taste perception. Gene duplication has happened in *T2R*s frequently, and what will be the fate of *T2R*s copies is another question we are concerned about. To answer these questions, we selected the *T2R*s of primates reported in another study and conducted corresponding selective pressure analyses to determine what kind of selective pressure was acting on them. Further, we carried out selective pressure analyses on gene copies and their corresponding ancestors by considering several possible situations. The results showed that among the 25 gene groups examined here, 15 groups are subject to purifying selection and others are under relaxed selection, with many positively selected sites detected. Gene copies existed in several groups, but only some groups (clade1_a1‐b2, clade1_c‐c2, clade1_d1‐d3, clade1_f1‐f2, *T2R10*, *T2R13*, and *T2R42*) have positively selected sites, inferring that they may have some relation to functional divergence. Taken together, *T2R*s in primates are under diverse selective pressures, and most gene copies are subject to the same selective pressures. In such cases, the copies may be just to keep the function conservative, and more copies can increase the quantity of the bitter taste receptor, raise the efficiency of bitter substance recognition, and finally enhance the fitness of feeding during the evolutionary course of primates. This study can improve our understanding of *T2R*s evolution in primates.

## Graphical abstract

We examined the selective pressure acting on the T2Rs of primates and tested whether the different copies of T2Rs have functional divergence. The result showed that 15 groups are subject to purifying selection while 10 groups are under relaxed selection, and although gene copies exist in several groups, only some groups have signatures of positive selection, indicating that the gene copies have functional divergence. In some groups, the copies may be just to keep the function conservative, and more copies can increase the quantity of the bitter taste receptor, raise the efficiency of bitter substance recognition, and finally enhance the fitness of feeding during the evolutionary course of primates.

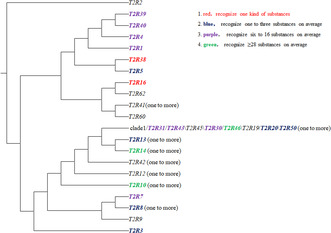

## 1 INTRODUCTION

Bitter taste perception has played an important role in protecting animals from consuming poisonous and harmful substances, and it happens through the interaction between the bitter tastant and the bitter taste receptor encoded by *T2R* genes. The relationship between gene repertoire size and feeding ecology has been reported in fish (Shang et al., 2017), reptiles (Zhong et al., 2017), birds (Behrens et al., 2014; Davis et al., 2010; Wang & Zhao, 2015), and mammals (Li & Zhang, 2014), and also *T2R* gene repertoire size is likely related to the special feeding habits or behaviors suggested in whales (Feng et al., 2014) and other mammals (Liu et al., 2016). Besides, ligands for some T2Rs are reported, and many of them are from plants. For example, T2R7 can recognize strychnine from plants of the genus *Strychnos* (Sainz et al., 2007), T2R14 can respond to noscapine from Papaver spp. (Behrens et al., 2004), T2R16 can detect salicin found in Salix spp. (Bufe et al., 2002), and T2R38 can react to isothiocyanates from the plants of the Brassicaceae family (Wooding, 2011; Wooding et al., 2010). The fact that T2Rs can recognize plant chemicals, and plants are the essential food source of most primates, suggests that bitter or poisonous material from plants can act as a driving force for the *T2R*s evolution of herbivorous primates (Wooding, 2011).

In regards to primates, the number of *T2R*s expanded (Hayakawa et al., 2014). Fischer et al. (2005) explored the evolution of bitter taste receptors in humans and apes and found that the mean d*N*/d*S* ratio of *T2R*s is not significantly different from one, although the d*N*/d*S* ratios of seven *T2R*s are bigger than one. Shi et al. (2003) compared the *T2R*s of humans and mice and found that both one‐to‐one gene orthology and species‐ or lineage‐specific gene duplications exist between humans and mice *T2R*s. Gene duplication is common (Zhang, 2003), and in humans, the duplicated genes even accounted for more than 70% (Kuzmin et al., 2022). Gene duplication indicates an important evolutionary mechanism for ecological adaptation by producing new hereditary material and different biological function (Jiao et al., 2018; Ohno, 1970; Zhang, 2003). The gene repertoires of vertebrates *T2R*s range from 0 to 219 (Feng et al., 2014; Li & Zhang, 2014; Zhong et al., 2021), and the gene repertoires of all organisms are current snapshots of selection and drift imposing on gene duplication events (Wacholder & Carvunis, 2021). In addition, the functional redundancy of duplicated genes is evolutionarily steady, and the duplicated genes can acquire functional specialization due to some specific structural and functional factors (Kuzmin et al., 2022). It is suggested that some lineages of *T2R*s in humans and mice are from species‐specific gene duplications (Shi et al., 2003), and gene duplications were particularly obvious in the ancestral branches of anthropoids (the anthropoid cluster) (Hayakawa et al., 2014); furthermore, multiple copies have been found in *T2R16* and *T2R41* in bats (Jiao et al., 2018), and an abundance of gene duplication events have been reported in the Euarchontoglires clade (Hayakawa et al., 2014). It is also suggested that a single‐copy gene evolves conservatively because it is subject to strong negative selection (Hughes & Criscuolo, 2008). Gene duplications generate an extra gene copy and thus alleviate the negative selection of one or both copies (Hughes & Criscuolo, 2008). Following gene duplication, the homologous regions (e.g., protein motifs or protein domains) of two duplicates may evolve at distinct rates due to the different constraints caused by functional divergence (Huang & Golding, 2012), and the existence of positively selected sites can be used as one way to account for such functional divergence (Strain & Muse, 2005).

The ratio (*ω*) of nonsynonymous to synonymous substitution rates (d*N*/d*S*) was used to estimate the selective pressure acting on the gene during the long‐term evolution through the codeml program in PAML. *ω*>, <, and =1 indicate positive selection, purifying selection, and neutral evolution, respectively.

Gene duplication frequently happens in *T2R* genes. Analysis of positive selection in the *T2R* gene family can provide an insight into understanding the evolution of duplicated genes. After duplication, one of the gene copies can diverge and gain new functions (Ohno, 1970). The positively selected sites can be used as a proxy to explain such functional divergence (Strain & Muse, 2005), and such sites are detected by the improved branch‐site model (Zhang et al., 2005), which can be used to examine, that along with species‐specific gene duplication, whether positive selection has acted on some additional sites. In this model, the foreground branches were set as all branches connecting to specific species (alternative model); the corresponding null model was the same as the alternative model, except that *ω* of the foreground branches was fixed at 1 (Wang et al., 2019; Yang & Nielsen, 2002; Zhang et al., 2005). Wang et al. (2019) used the improved branch‐site model to examine whether the hummingbird‐specific *T2R1* duplicates have experienced positive selection and detected 5.7% sites with positive selection signature, and then they revealed new functions in the hummingbird *T2R* gene copies resulted from a lineages‐specific duplication, shaped by positive selection. Such a method was also used to detect positively selected sites in mice and bats, and the functional divergence of duplicated *T2R*s was reported (Jiao et al., 2018; Lossow et al., 2016).

Primates are a group of mammals that display exceptional ecological and dietary diversity (Fleagle, 1999), and previous studies have investigated *T2R*s evolution in humans, all extant apes (chimpanzees, bonobos, gorillas, and orangutans), rhesus macaques, and baboons (Fischer et al., 2005; Hayakawa et al., 2014). According to Zhang (2003) and Magadum et al. (2013), the fate of duplicated gene can be pseudogenization, conservation of gene function, subfunctionalization, and neofunctionalization. In primates, *T2R* gene duplication occurs frequently, and some species even possess six *T2R*s duplicates in one cluster (P.F., H.W., X.L., X.D., Q.L., F.S., Q.Z., unpublished data). The increased availability of *T2R*s sequences in the published allows for the investigation of the molecular evolution of the *T2R* gene family and the evaluation of selection following duplication events, and the frequently occurring gene duplicate cases provide an opportunity to predict if there are functional divergences among the genes after duplication. As the function of human T2Rs is identified and categorized into four groups, and it is also suggested that non‐human primates can share the response character with their human orthologs (Behrens et al., 2014; Bufe et al., 2002), we related the primates bitter taste receptors' function to the character of their human orthologs.

Thus, the aims of our present study are to (1) determine the selective pressures (indicated by d*N*/d*S*) acting on primates *T2R*s and relate them to the function; (2) and evaluate whether the *T2R* copies, especially the lineage‐specific *T2R* copies generated by gene duplication, are under positive selection. Finally, we tested the normality of d*N*/d*S* distributions by using a Kolmogorov–Smirnov test. If the normality cannot be rejected (*p* > .05), a one‐sample *t*‐test will be conducted to examine if the mean values are significantly different from one another (Fischer et al., 2005). This study is an expanded research of previous studies which investigated the location of positively selected sites and its relation to the function (Dong et al., 2021; Wooding, 2011) the bitter tastant profile of human (Meyerhof et al., 2010), the *T2R*s repertoire sizes of Euarchontoglires (Hayakawa et al., 2014), d*N*/d*S* of *T2R*s in human (Wang et al., 2004), and functional diversity or divergence of T2R16 in several primates (Imai et al., 2012; Itoigawa et al., 2021); and the difference between the above mentioned research and our study lies in that we concerned on the d*N*/d*S* and the functional divergence prediction of *T2R*s in primates from different orders, further explored the significance of *T2R*s duplication in primates.

## 2 MATERIALS AND METHODS

### 2.1 Data sources

The sequences of *T2R*s are collected from https://doi.org/10.5061/dryad.r7sqv9sg9, which was submitted previously by our team after data mining. These sequences are from the genomes of primates, which include 16 species from Cercopithecidae, five species from Hominidae, four species from Cebidae, three species from Lemuridae, and another six species. The species and *T2R* gene names are listed in Table S1.

### 2.2 Sequence alignment and phylogenetic reconstruction

The resulting sequences were aligned with MEGA 6 (Tamura et al., 2013) and checked by eye. The alignments of nucleotide sequences were obtained according to protein sequence alignments and were subsequently used for selective pressure analyses.

The phylogenetic tree of each gene cluster was reconstructed by both neighbor‐joining (NJ; Saitou & Nei, 1987) and ML approaches supplemented in MEGA6, with mouse *V1RE9* (accession No. AF454731) as the outgroup gene. The NJ tree was reconstructed by using the settings as follows: the Kimura two‐parameter model (Nei & Kumar, 2000) was used; the gaps/missing data was treated by pairwise deletion; and the number of bootstrap replicates was set to 1000 (Felsenstein, 1985). In ML tree reconstruction, the general time reversible model was used. Meanwhile, we also referred to the tree reconstructed by Mrbayes, which was provided in one of our previous research (Feng et al., under review).

### 2.3 Evolutionary analysis of each gene clade/cluster

To understand the evolution of each gene, we performed the evolutionary analyses on each gene clade/cluster using PAML4.9 by estimating the ratio of nonsynonymous to synonymous substitution rates (*ω*), which is an indicator of selective pressure. To understand the evolution of *T2R*s and to predict whether different copies of a gene have functional divergences, we conducted a series of selective pressure analyses on the gene sequences. In this section, the branch model, site model, and branch‐site model were used. The branch models allowing *ω* to vary along the branches were used to detect positive selection imposed on specific lineages. The site models were used to discover the positively selected sites among different sites (Song et al., 2013). The improved branch‐site model (Zhang et al., 2005) analyses were conducted to check whether positive selection was imposed on additional sites along with gene duplication (Wang et al., 2019). In addition, a likelihood ratio test (LRT) was conducted to examine which model fits the data better and to detect a positive selection signature when carried out the branch model, site model, or branch‐site model analyses by comparing twice the log likelihood difference between the pairs of models with a chi‐square distribution, and the differences between model parameters were used as the degrees of freedom (Yang, 2000). Besides, the Bayes empirical Bayes (BEB) method (Yang et al., 2005) was used to estimate the posterior probability (PP) of positively selected sites.

In specific, first, we undertook branch model analyses in the codeml program in PAML by comparing the two models, Model M0 (one *ω*) and Model M0 (*ω* = 1), in which *ω* is allowed to be a single value across a gene, with *ω* of M0 (one *ω*) being any value while *ω* of M0 (*ω* = 1) being fixed to 1. This step aims to investigate the evolution of *T2R*s. After that, we tested the normality of d*N*/d*S* distributions by using a Kolmogorov–Smirnov test. If the normality could not be rejected (*p* > .05), a one‐sample *t*‐test will be conducted to examine whether the mean value is significantly different from one or not (Fischer et al., 2005). Second, gene clades/clusters are conducted site model analyses to detect the positively selected sites, respectively. The site models were tested comparatively (Anisimova et al., 2001): M1a (nearly neutral) versus M2a (positive selection), M7 (beta) versus M8 (beta & *ω* > 1) and M8 (beta & *ω* > 1) versus M8 (beta & *ω* = 1). In addition, branch model analyses were also conducted by comparing the one‐ratio model, assigning the same *ω* ratio to all branches along the tree, to the two‐ratio model, which assigned two *ω* ratios to the foreground (*ω*_1_) and background branches (*ω*_2_), respectively (Borges et al., 2012; Yang & Nielsen, 2002). Third, if the gene has two or more copies, the branch model and improved branch‐site model, which set the branches leading to two or more copies as foreground branches while others as background branches, were carried out and compared to identify the positively selected sites. Fourth, selective pressure analysis was carried out to test whether a significantly different *ω* exists between the common ancestor of two or more *T2R*s and other *T2R*s by assigning *ω*_1_ to the ancestral branch and *ω*_2_ to other branches.

## 3 RESULTS

### 3.1 The construction of phylogenetic gene tree and *T2R* gene groups

The phylogenetic gene tree constructed by Mega software through ML or NJ methods was compared with that from our previous study (Feng et al., under review), which reconstructed by Mrbayes (see details in Figure S1), and we found that most topologies of the trees are similar but the bootstrap values of Mrbayes tree are high; thus, we used the Mrbayes tree as the guide tree to perform selective pressure analyses (Figure S1).

**FIGURE 1:**
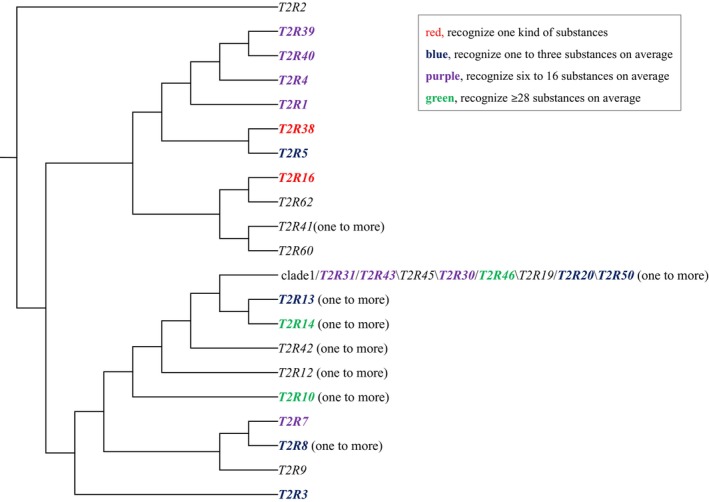
Clusters and their orthologous human *T2R*s. The receptors that have been investigated by Meyerhof et al. (2010) are indicated by different colors. Among them, red color indicates that the receptor can recognize one kind of substance; blue color means the receptors can respond to one to three substances on average; purple color denotes that the receptors can react to 6–16 bitter tastants on average; green color suggests that the receptors can recognize ≥28 substances on average. Besides, clusters contain one or more orthologs are marked.

**FIGURE 2:**
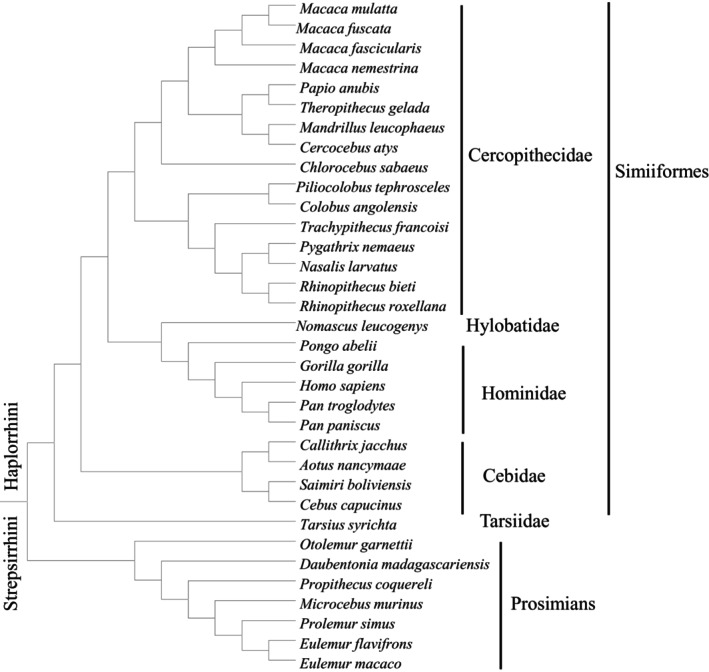
The phylogeny of species used in this study. Adjusted from Perelman et al. (2011).

In the Mrbayes tree, these *T2R*s mainly formed 21 clusters, and each cluster consisted of 10–51 genes. We named them according to the human orthologous gene clustered with them, or their relationship with the corresponding human pseudogene. The result showed that some gene clusters (*T2R1*, *T2R2*, *T2R3*, *T2R5*, *T2R4*, *T2R7*, *T2R9*, *T2R16*, *T2R38*, *T2R39*, *T2R40*, *T2R60*, *T2R62*) are one‐to‐one orthologous genes, while others (clade1, *T2R8*, *T2R10*, *T2R12*, *T2R13*, *T2R14*, *T2R41*, *T2R42*) are one to more orthologous genes that include two or more gene copies, and we summarized them in Figure 1. Especially, clade1 has several inner clades, and we categorized them as clade1_a1‐b2, clade1_c‐c2, clade1_d1‐d3, clade1_e1‐e2, and clade1_f1‐f2. It is suggested that some non‐human primates' receptors share the reaction characters with human orthologs in responding to bitter substances (Behrens et al., 2014; Bufe et al., 2002). Thus, we also referred to the human T2Rs and marked the numbers of their bitter tastants, which were tested in Meyerhof et al. (2010) for the following analyses (Figure 1). In Figure 1, several clusters have more than one copy, and we marked them with “one to more.” That is, in clade 1, many Cercopithecidae species have two gene copies; in the *T2R8* cluster, *Callithrix jacchus* has two copies: Caja_800_529, Caja_320_049; in the *T2R10* cluster, *Otolemur garnettii* has five copies: Otga_548_561, Otga_767_792, Otga_012_932, Otga_727_641, Otga_707_633; in the *T2R12* cluster, *Eulemur flavifrons*, *Eulemur macaco*, and *Microcebus murinus* each have two copies: Eufl_055_993, Eufl_408_225; Euma_055_993, Euma_370_087; Mimu_304_042, Mimu_121_838; in the *T2R13* cluster, *O. garnettii*, *Daubentonia madagascariensis*, *M. murinus*, and *Prolemur simus* each have two copies: Otga_079_134, Otga_942_997; Dama_138_049, Dama_406_317; Mimu_791_502, Mimu_706_417; Prsi_961_872, Prsi_598_509; in the *T2R14* cluster, *Papio anubis* and *Tarsius syrichta* each have two copies: Paan_892_851, Paan_086_045; Tasy_742_492, Tasy_762_521; in the *T2R41* cluster, *T. syrichta*, *Propithecus coquereli*, and *O. garnettii* each have two copies: Tasy_659_385, Tasy_594_320; Prco_772_498, Prco_739_465; Otga_004_116, Otga_603_712; in the *T2R42* cluster, *Pongo abelii*, *T. syrichta*, and *E. macaco* each have two copies: Poab_467_411, Poab_430_344; Tasy_901_642, Tasy_225_966; Euma_979_920, Euma_580_500; and *M. murinus* has six copies: Mimu_028_766, Mimu_367_084, Mimu_690_422, Mimu_960_692, Mimu_769_510, Mimu_376_105; while *P. simus* and *P. coquereli* each have four copies: Prsi_734_666, Prsi_422_363, Prsi_666_586, Prsi_936_871; Prco_078_816, Prco_588_293, Prco_683_388, Prco_917_649, for details please see Table S1. In addition, the phylogeny of primates used in this study is summarized in Figure 2.

### 3.2 The evolution of *T2R* genes

We conducted the evolutionary analyses of *T2R*s on each gene cluster/clade and found that the selective pressures (*ω*) of some genes (*T2R2*, *T2R3*, *T2R4*, *T2R5*, *T2R7*, *T2R8*, *T2R10*, *T2R38*, *T2R39*, *T2R40*, *T2R41*, *T2R42*, clade1_e1‐e2, *T2R60*, *T2R62*) are significantly lower than 1 (*p*‐value < .05), thus they are subject to purifying selection; while in other genes (*T2R1*, *T2R9*, *T2R16*, *T2R12*, *T2R13*, *T2R14*, clade1_a1‐b2, clade1_c‐c2, clade1_d1‐d3, clade1_f1‐f2), the *ω* is not significantly different from 1 (Table 1), indicating that the pressure of purifying selection acting on these genes is generally relaxed. To compare our results with those of the previous, we collected and listed the results of Fischer et al. (2005) and Wang et al. (2004) in Table 1. The result showed that the *ω* of our study is roughly consistent with that of the previous.

**TABLE 1:** *ω* of each clade and the previous study.

	*p*‐Value of M0 vs. M0 (*ω* = 1)	*ω*	*ω* of study^a^	*ω* of study^b^
*T2R1*	.27	0.92	0.6	0.82
*T2R2*	**1.31E‐14**	**0.50**	NA	NA
*T2R3*	**1.72E‐06**	**0.66**	0.59	0.21
*T2R4*	**1.69E‐03**	**0.75**	0.88	0.43
*T2R5*	**.02**	**0.78**	0.91	0.96
*T2R7*	**1.13E‐15**	**0.45**	0.5	0.82
*T2R8*	**1.76E‐04**	**0.72**	0.89	NA
*T2R9*	.87	0.98	0.86	0.54
*T2R10*	**.04**	**0.86**	0.81	NA
*T2R12*	.12	0.85	NA	NA
*T2R13*	.39	1.07	1.58	2.14
*T2R14*	.46	1.06	1.11	0.53
*T2R16*	.30	0.92	0.81	0.20
clade1_f1‐f2 (*T2R19*/*T2R20*)	.74	1.03	1.48/1.48	NA/NA
clade1_a1‐b2 (*T2R30*/*T2R46*)	.32	1.11	1.22/0.96	NA/0.78
*T2R38*	**3.75E‐11**	**0.54**	0.43	0.28
*T2R39*	**4.26E‐07**	**0.54**	0.76	0.79
*T2R40*	**4.06E‐13**	**0.51**	0.75	0.80
*T2R41*	**8.55E‐06**	**0.72**	0.85	2.14
*T2R42*	**.02**	**0.88**	1.11	0.95
clade1_c‐c2 (*T2R31*/*T2R43*)	.23	0.87	1.23/0.95	0.66/0.78
clade1_d1‐d3 (*T2R45*)	.92	0.99	NA	1.01
clade1_e1‐e2 (*T2R50*)	**.04**	**0.82**	0.87	2.01
*T2R60*	**4.47E‐06**	**0.65**	0.9	3.34
*T2R62*	**4.73E‐04**	**0.77**	NA	NA

*Note*: *p*‐Value < .05 and its corresponding *ω* are marked in bold.

^a^ The result is from Fischer et al. (2005). Their average is not significantly different from one (one‐sample *t*‐test, *p* = .22).

^b^ The result is from Wang et al. (2004), and none of them are significantly different from 1.

For the one‐to‐one orthologous genes, we next examined the positively selected sites of *T2R*s through site models and found that seven gene clusters (*T2R1*, *T2R3*, *T2R4*, *T2R5*, *T2R7*, *T2R16*, and *T2R62*) have positively selected sites identified by both the M2a and M8 models (Table 2 and Table S2). For many sequences, the positions are referred to as human homologous sequences, and if no human homologous sequences existed in the cluster, we used the sequences of *Pan troglodytes* (*T2R2* cluster, *T2R62* cluster) or *O. garnettii* (*T2R12* cluster) as the references. It is worthy to mention that, in the *T2R38* cluster, M7 versus M8 are significantly different, with a positively selected site detected, but in the *T2R39* cluster and the *T2R40* cluster, no positively selected sites are detected, no matter whether M7 versus M8 are not significantly different (the *T2R39* cluster) or significantly different (the *T2R40* cluster).

**TABLE 2:** The positively selected sites detected in the one‐to‐one orthologous gene cluster.

Gene	Positively selected sites identified by M2a and M8 models (BEB)^a^
*T2R1*	77 M, 80 A, 83 A, 85 L, 86 L, 150 F, 167 T, 168 L, 202 R, 241 V, 254 I
*T2R3*	74 T, 145 A, 271 T
*T2R4*	3 R, 70 V, 74 T, 75 E, 85 V, 264 L
*T2R5*	142 Q, 146 T, 149 H, 171 Q
*T2R7*	209 R
*T2R16*	61 A, 69 S, 178 T
*T2R62*	75 P, 136 E, 149 Q, 166 V, 289 Q, 295 D, 296 A

^a^ The sites are under the model comparison of M1a vs. M2a, M7 vs. M8, with *p*‐value < .05, and posterior probability (PP) >95%.

For clusters that have two or more gene copies, the site model and the improved branch‐site model were used to detect positively selected sites. The improved branch‐site model was used by comparing modified model A with the corresponding null model with *ω*_2_ = 1 fixed (fix_omega = 1 and omega = 1), and the positively selected sites identified by both site models (M2a and M8) and the improved branch‐site model were summarized in Table 3. The results showed that some duplicated genes (*T2R10*, *T2R13*, clade1_a1‐b2, clade1_c‐c2, clade1_d1‐d3, clade1_f1‐f2, *T2R42*) have positively selected sites while others not (Tables S2 and S3).

**TABLE 3:** The positively selected sites identified by both site models and the improved branch‐site model (BEB) in the clade/cluster which have two or more gene copies.

Gene	Positively selected sites identified by both site models and the improved branch‐site model (BEB)	*p*‐Value of null model vs. alternative model
clade1_a1‐b2 (*T2R30*/*T2R46*)	89 A, 99 A, 210 L, 243 S, 255 K, 262 C, 266 I, 291 V	**5.13E‐21**
clade1_c‐c2 (*T2R31*/*T2R43*)	295 Q	**7.39E‐04**
clade1_d1‐d3 (*T2R45*)	151 Q, 154 W, 183 A, 296 Q	**5.55E‐10**
clade1_f1‐f2 (*T2R19*/*T2R20*)	80 R, 251 L	**4.10E‐05**
*T2R10*	68 Q	**2.69E‐07**
*T2R13*	158 R, 179 K, 270 V	**1.74E‐08**
*T2R42*	65 D, 90 H, 167 D, 254 I, 258 M	**1.54E‐14**

*Note*: *p*‐Value < .05 is marked in bold.

Further, we tested whether the ancestor of the duplicated gene has been subjected to stricter or looser selective pressure. In details, in the *T2R10* cluster, *O. garnettii* has five copies, and we wondered whether the ancestor of *O. garnettii* or prosimians has experienced particular selective pressures. Thus a two‐ratio model by setting the branch leading to prosimians or four *O. garnettii* genes was tested and compared with a one‐ratio model, which allows all the branches to have one *ω*. As a result, no significant difference in *ω* was observed in these two pairs of model comparison. In addition, two *T. syrichta T2R*s also existed in the *T2R10* cluster. We conducted a two‐ratio model by setting the two *T. syrichta T2R*s as foreground while others as background, and then a two‐ratio model was compared with the one‐ratio model and found that two *T. syrichta T2R*s were under much stronger purifying selection than others (*ω*_1_ = 0.53, *p*‐value < .01). In the *T2R13* cluster, *P. simus*, *M. murinus*, *D. madagascariensis*, and *O. garnettii* each have two copies. We tested what kind of selective pressure is the ancestor of prosimians subject to by setting the ancestral branch leading to prosimians as the foreground while the rest as the background, and the result showed that the ancestral branch is under positive selection (*ω*_1_ = 3.71, *p*‐value = .03), indicating that the ancestor gene has experienced positive selection and then diverged into the descendant genes which are subject to selective constraint relaxation; but when considering the ancestor of simians (New World monkeys, Old World monkeys and apes) by using the same methods, we found that it is subject to purifying selection (*ω*_1_ = 0.49, *p*‐value = .02), suggesting that the ancestor gene of simians' *T2R*s is conservative. In the *T2R14* cluster, *P. anubis* and *T. syrichta* each have two *T2R* gene copies, but in *P. anubis*, the two copies are identical although they are distributed in different scaffolds, and *ω* of *T. syrichta* (*ω*_1_ = 0.74) was significantly different from others (*ω*_2_ = 1.14, *p*‐value = .04) when comparing one ratio with two ratios that assigned the branches leading to *T. syrichta* as the foreground branch and the rest as the background branch, suggesting stronger selective pressure on *T2R*s of *T. syrichta* (Table 4). In the *T2R41* cluster, *O. garnettii*, *P. coquereli*, and *T. syrichta* each have two copies. The model comparison suggested that the branch leading to *T. syrichta* is subject to much stronger purifying selection than do others (*ω* = 0.34, *p*‐value = .03). In the *T2R42* cluster, *M. murinus* has six *T2R*s copies, while both *P. simus* and *P. coquereli* have four *T2R*s copies, and *T. syrichta* has two copies. We first tested the one ratio in this clade and tested the two‐ratio model by assigning the branch leading to prosimians as the foreground and others as the background. We found that the branch leading to prosimians is subject to purifying selection, and it is under much stronger selective pressure than do other branches (*ω* = 0.51, *p*‐value = .04), indicative of the functional importance of *T2R*s in the ancestry of prosimians.

**TABLE 4:** Likelihood ratio tests of selective pressures on the clade/cluster with gene copies.

Models	*ω* (d*N*/d*S*)	Comparisons	*p*‐Value
*T2R10* cluster: 36 *T2R* gene sequences (*Otolemur garnettii* has five copies and *Tarsius syrichta* has two *T2R* gene copies)			
A. All branches have the same *ω*		B vs. A	**<.01**
B. The branches of two Tasy *T2R*s has *ω*_1_, and others have *ω*_2_	*ω*_1_ = 0.53, *ω*_2_ = 0.95		
*T2R13* cluster: 40 *T2R* gene sequences (*Prolemur simus*, *Microcebus murinus*, *Daubentonia madagascariensis*, *O. garnettii* each have two copies)			
A. All branches have the same *ω*	*ω* = 1.07		
B. The ancestral branch leading to prosimii species has *ω*_1_ and other branches have *ω*_2_	*ω*_1_ = **3.71**, *ω*_2_ = 1.03	B vs. A	**.03**
*T2R14* cluster: 31 *T2R* gene sequences (*Papio anubis* and *T. syrichta* each have two *T2R* gene copies)			
A. All branches have the same *ω*	*ω* = 1.06	B vs. A	**.04**
B. The branches leading to *T. syrichta* have *ω*_1_ and other branches have *ω*_2_	*ω*_1_ = 0.74, *ω*_2_ = 1.14		
*T2R41* cluster: 36 *T2R* gene sequences (*O. garnettii*, *Propithecus coquereli*, and *T. syrichta* each have two *T2R* gene copies)			
A. All branches have the same *ω*	*ω =* 0.72	B vs. A	**.03**
B. The branch leading to *T. syrichta* has *ω*_1_, and others have *ω*_2_	*ω*_1_ = 0.34, *ω*_2_ = 0.75		
*T2R42* cluster: 44 *T2R* gene sequences (*M. murinus* has six *T2R*s copies while both *P. simus* and *P. coquereli* have four *T2R*s copies, and the *T. syrichta* has two copies)			
A. All branches have the same *ω*			
B. The branch leading to prosimians has *ω*_1_, and others have *ω*_2_	*ω*_1_ = 0.51, *ω*_2_ = 0.90	B vs. A	**.04**
Clade1_c‐c2 (*T2R31*/*T2R43*): 36 *T2R*s sequences (The sequence of Cercopithecidae species nearly have two copies)			
A. All branches have the same *ω*	*ω* = 0.87	B vs. A	**.04**
B. The branch of all the Cercopithecidae species have *ω*_1_ and six other primates genes has *ω*_2_	*ω*_1_ = 0.76, *ω*_2_ = 1.31		

*Note*: *p*‐Value < .05 is marked in bold.

For the clades that contain gene duplication mainly from the haplorrhine species, we found that in the Cercopithecidae species, gene duplication has occurred in the whole clade other than only in one or two genes, as does in prosimians. In clade1_a1‐b2, the genes of clade a1 and a2, clade b1 and b2, are two copies, respectively. We tested whether the branch leading to Cercopithecidae has experienced accelerated evolution or not by conducting a two‐ratio model, assigning *ω*_1_ to it and others *ω*_2_. However, when comparing two ratio with one ratio allowing all the branches to have a single *ω*, we found that no significant difference existed between these two models, although the *ω* for the branch leading to Cercopithecidae species is bigger than that of other branches (*ω* = 3.13), indicating that the *ω* for the Cercopithecidae branch is not different from that of others. In clade1_c‐c2, nearly all the Cercopithecidae species have two *T2R*s gene copies, and we conducted the one‐ratio and two‐ratio model analyses, which allowed all the branches to have one *ω*, and the genes of Cercopithecidae species have *ω*_1_ while others have *ω*_2_, respectively. The results showed that the selective pressures acting on Cercopithecidae species are different from others (*p*‐value = .04). Such analyses were also conducted on clade1_d1‐d3, clade1_e1‐e2, clade1_f1‐f2, and other gene cluster that have gene duplication, but no significant differences were detected. Only the cases with significant differences are listed in Table 4.

Taken together, we conducted selective analyses in the clades/clusters that have gene duplication and found that positively selected sites existed in genes from clade1_a1‐b2, clade1_c‐c2, clade1_d1‐d3, clade1_f1‐f2, *T2R10* cluster, *T2R13* cluster, and *T2R42* cluster, while in other clusters no positively selected site was detected. But in the ancestor of duplicated gene, only the ancestor of the *T2R10* cluster, *T2R13* cluster, *T2R14* cluster, *T2R41* cluster, *T2R42* cluster, and clade1_c‐c2 has different selective pressure from that of their decendant gene, respectively.

## 4 DISCUSSION

In this study, we examined the evolution of *T2R*s in primates and predicted whether the copies of different *T2R*s have functional divergence based on selection analyses. The results showed that among the 25 gene groups, some are subject to purifying selection while others are under relaxed selective constraints. Among the genes for which *ω* is not significantly different from 1, the *ω* for *T2R1*(0.92), *T2R9*(0.98), *T2R12*(0.85), *T2R16*(0.92), clade1_c‐c2(0.87), and clade1_d1‐d3(0.99) is less than 1, indicative of relaxation of selection, while in *T2R13*(1.07), *T2R14*(1.06), clade1_a1‐b2(1.11), and clade1_f1‐f2(1.03), the *ω* is greater than 1, which suggests more nonsynonymous than synonymous mutations are accumulated in these genes. In the one‐to‐one orthologous genes, seven genes (*T2R1*, *T2R3*, *T2R4*, *T2R5*, *T2R7*, *T2R16*, and *T2R62*) have positively selected sites identified from both M2a and M8 models. In other cases, positively selected sites were also detected by M2a and M8, even in the improved branch‐site models. Such sites are likely linked to the critically functional regions of the receptors. Just as it was found in the previous studies, for example, in T2R1, the positively selected sites are concentrated on the first and second extracellular loops (EL1, EL2) involved in ligand binding (Dong et al., 2021). In T2R16, the positively selected sites are fewer, which may be related to primates. T2R16 specifically recognizes β‐glucosides, and a previous study found that the low sensitivity to β‐glucosides in T2R16 of bamboo lemurs has accounted for their high‐cyanide bamboo consumption (Itoigawa et al., 2021). Hu et al. (2020) tested the function of T2R49 (T2R20), of which signatures of positive selection were detected in pandas, and this gene has been directionally selected at A52V and Q296H in the panda of the Qinling population (Hu et al., 2020; Zhao et al., 2013), and found that the receptor with two variants, A52V and Q296H, confers a remarkedly lower sensitivity to quercitrin when compared with pandas with other variants. Besides, Wang et al. (2019) demonstrated new functions of hummingbird *T2R* gene copies that originated from duplication, shaped by positive selection. The exact functions of the positively selected sites detected in this study still need further exploration in the future.

Among the genes, 15 of which (*T2R2*, *T2R3*, *T2R4*, *T2R5*, *T2R7*, *T2R8*, *T2R10*, *T2R38*, *T2R39*, *T2R40*, *T2R41*, *T2R42*, *T2R50*, *T2R60*, and *T2R62*) are subject to purifying selection with *ω* = 0.45–0.88, significantly different from 1 (*p*‐value < .05); in other genes, the range of *ω* is from 0.85 to 1.11, and none of them are significantly different from 1. In contrast, Wang et al. (2004) examined the intra‐specific variations of all 25 human genes, and found that although some *ω*s of the 25 human *T2R* genes appeared much higher than others, none of them were significantly different from 1. Besides, Fischer et al. (2005) investigated the T2R of humans and all extant ape species and found that the overall d*N*/d*S* ratios for the entire tree for each gene varied between 0.43 and 1.58, and their average was not significantly different from one (one‐sample *t*‐test, *p* = .22). However, in our study, the results showed that the selective pressure for each gene is from 0.45 to 1.11, and the mean d*N*/d*S* ratio is significantly different from one (one‐sample *t*‐test, *p*‐value < .01). That is, our study is different from the results reported by Wang et al. (2004) and Fischer et al. (2005) to some extent. This is probably due to the difference in samples. In Fischer et al. (2005), seven species, including five from Hominidae and two from Cercopithecidae, were used, and in Wang et al. (2004), two species from Hominidae were used. However, in our study, 1 species from Tarsiiformes, 5 species from Lemuriformes, 1 species from Lorisformes, and 27 species from old world and new world monkeys, especially 16 species from the Cercopithecidae, were used. The species in Cercopithecidae mainly feed on plants, which contain an abundance of bitter substances; therefore, it is necessary to maintain the bitter taste function for the Cercopithecidae species and thus likely result in the purifying selection of some *T2R*s.

When further compared with Wang et al. (2004) and Fischer et al. (2005), we found that the results of our study were more similar to those of Fischer et al. (2005) than did Wang et al. (2004). The genes under purifying selection are *T2R3*, *T2R4*, *T2R5*, *T2R7*, *T2R16*, *T2R39*, and *T2R40*, which are also supported by Fischer et al. (2005) and Wang et al. (2004). However, in *T2R41*, *T2R50*, and *T2R60*, both Fischer et al. (2005) and our study suggest that they are under purifying selection, while in Wang et al. (2004) they are under positive selection. Fischer et al. (2005) and the present study sampled more widely than that of Wang et al. (2004). Specifically, Fischer et al. (2005) used five primates from Hominidae and two primates from Cercopithecidae, and the primates of our present study encompass 34 species from Haplorrhine and Strepsirrhini. In contrast, Wang et al. (2004) used a very limited number of species, including one chimpanzee and 22 unrelated humans. Thus, it is likely that the sample unevenness contributed to this difference, and also because Wang et al. (2004) are focused on humans, it is reasonable that the evolutionary trends are different between modern human from wild primate species.

The evolutionary ratios among the genes are different, from 0.54 to 1.11, indicating that the selective pressure acting on them is differentiated, which may be related to function differentiation. The previous study found that some receptors are narrowly tuned, for example, hT2R3 and hT2R5, which reacted merely to a single compound, the antimalarial drug chloroquine and 1,10‐phenanthroline, respectively; in contrast, some bitter receptor can respond to many chemicals, such as hT2R14, which recognized 33 compounds of the 104 substances tested, and some T2Rs respond to a medium‐sized amount of bitter substances (Meyerhof et al., 2010). In other species, the receptors of rodent and zebra finch are narrowly tuned, and the T2Rs of chicken and turkey are broadly tuned, whereas those of frogs are tuned from broadly to narrowly (Behrens et al., 2014; Bufe et al., 2002). Some T2Rs names in Meyerhof et al. (2010) are different from those in our study, and the T2R44, T2R47, T2R48, and T2R49 in Meyerhof et al. (2010) correspond to T2R31, T2R30, T2R19, and T2R20 in our study. We compared the evolutionary rate of T2Rs according to their tuning width and found that the T2Rs that recognized less bitter substances are prone to suffer from purifying selection. For example, the T2Rs in group 4 (containing hT2R10, hT2R14, and hT2R46) can recognize many bitter substances, and one out of three T2Rs is under purifying selection; in group 2 (consisting of hT2R3, hT2R5, hT2R8, hT2R13, hT2R49/T2R20, and hT2R50), of which the receptors are narrowly tuned, the percentage of purifying selection is two thirds, suggesting a more important role for these receptors. Although the d*N*/d*S* of *T2R2*, *T2R9*, *T2R12*, *T2R19*, *T2R41*, *T2R42*, *T2R45*, *T2R60*, and *T2R62* is also obtained, they aren't classified into any of the groups mentioned above because no corresponding tastants were detected for them in Meyerhof et al. (2010). Several orphan genes (*T2R9*, *T2R41*, *T2R42*, *T2R45*, and *T2R60*) and a pseudogene (*T2R62*) in humans are under purifying selection here, which is likely attributed to the importance of their function in non‐human primates. Especially humans can avoid poisonous or bitter food through cooking or culture learning, which eases the burden of T2Rs to some extent, whereas non‐human primates cannot do that.

Another topic we focused on was functional divergence prediction between/among the gene copies. There are 11 clades with two or multiple gene copies, and only in the *T2R13* cluster is the ancestor of prosimians subject to positive selection; the genes from clade1_c‐c2 are from the species of Cercopithecidae and Hominidae, and the result of the likelihood ratio test (LRT) between one ratio and two ratio showed that in Hominidae, the genes are under positive selection (*ω*_2_ = 1.31); however, the two copies of the Cercopithecidae gene are subject to purifying selection (*ω* = 0.76). This is likely because Cercopithecidae mainly feed on plants containing more bitter substances, and the purifying selection can keep the T2Rs conservative to deal with the plenty of plants encountered. The *T2R42* cluster consists of T2Rs from the prosimians, and most of them have multiple copies. We tested one ratio, which assigned all branches to one *ω*, and two ratio, allowing the branch leading to prosimians to have *ω*_1_, and others *ω*_2_. The result of LRT between one ratio and two ratio showed that the ancestor is subject to much stronger purifying selection than others (*p* = .05, *ω*_1_ = 0.54, *ω*_2_ = 0.94), indicating that the T2Rs are under relaxed selection after diverging from their ancestor. In contrast, in the *T2R13* cluster, the ancestral gene of prosimians was subject to positive selection (*ω*_1_ = 3.71), and its descendant genes are under selective relaxation (*ω* = 1.07) after splitting from their ancestral gene. This is probable because the function of receptors in these two clusters are different. In specific, human T2R13, which detects very limited bitter tastants, clustered with the prosimians T2Rs in the *T2R13* cluster, and the overall d*N*/d*S* is 1.07. Considering that most prosimians mainly feed on insects, we speculated that the increase of d*N*/d*S* in the ancestor of prosimians T2Rs was due to the fact that the ancestor encountered more or specialized bitter substances during the feeding course. But in the *T2R42* cluster, all of the *T2R*s are from prosimians; that is, they are prosimians‐specific genes that may evolve to cope with prosimians‐specific foods that include special bitter substances; thus, their ancestor gene suffered from stronger purifying selection. In the *T2R41* cluster, *T. syrichta* has two *T2R* gene copies, of which the ancestor suffers from much stronger purifying selection (*ω*_1_ = 0.34) while the descendant genes are under accelerated evolution (*p* = .03, *ω*_2_ = 0.75). It is suggested that after gene duplication, the evolutionary rate of duplicated genes may increase in a short time as a consequence of the relaxed functional constraints after gene duplication and later will decline owing to the increase of functional constraints (Huang & Golding, 2012). However, in our study, the branch model analyses revealed that although multiple copies existed in clade1_a1‐b2/d1‐d3/f1‐f2, cluster *T2R8*/*T2R10*/*T2R14*, no putatively functional divergence was detected between/among the duplicated genes, and the d*N*/d*S* of some ancestral genes were not significantly different from that of the corresponding descendant genes, which may due to the short time of duplicated events, and another possible reason is that the redundant genes are only for increasing dosage to raise efficiency (Copley, 2020; Kitanovic et al., 2018), and such case conformed to the increased gene‐dosage advantage model, one of the mechanisms raised to interpret the preservation of gene duplicates in the genome (Konrad et al., 2011; Pegueroles et al., 2013).

In addition, feeding behavior and other factors may have an effect on the T2Rs evolution, such as using fire to cook (Liang et al., 2021; Wang et al., 2004), and the life experience passed from generation to generation can help animals avoid being poisoned by harmful substances. Furthermore, functional assay also revealed that some duplicated genes have functional divergences. Such assays were conducted on tandemly repeated T2Rs of monotremes (T2R810‐814) and T2R813 paralogs of platypus, and these receptors exhibit divergent response profiles (Itoigawa et al., 2022). A similar example of functional divergence among duplicated T2Rs can also be found in mice, bats, and hummingbirds (Jiao et al., 2018; Lossow et al., 2016; Wang et al., 2019). For the duplicated gene, with many positively selected sites identified in this study, we suggested that these sites may be related to the functional divergence of duplicated genes or play an essential role in recognizing bitter tastants. The function of the predicted sites and the functional divergence of duplicated receptors reported in this study are expected to be tested in further functional assay experiments.

## AUTHOR CONTRIBUTIONS

**Ping Feng:** Conceptualization (lead); formal analysis (lead); writing – original draft (lead); writing – review and editing (lead). **Xinyue Liang:** Data curation (equal); formal analysis (equal); visualization (equal). **Hongling Yu:** Data curation (equal); formal analysis (equal). **Xiaoyan Dong:** Investigation (equal). **Qiufang Liang:** Investigation (equal). **Chuanyin Dai:** Writing – review and editing (supporting).

## CONFLICT OF INTEREST STATEMENT

The authors have no conflicts of interest to declare.

## Supporting information


Figure S1.

Table S1.

## Data Availability

The sequences of *T2R*s are deposited at https://doi.org/10.5061/dryad.r7sqv9sg9, and other relevant data are presented in the paper and the appendix.

## References

[ece310610-bib-0001] Anisimova, M. , Bielawski, J. P. , & Yang, Z. (2001). Accuracy and power of the likelihood ratio test in detecting adaptive molecular evolution. Molecular Biology and Evolution, 18, 1585–1592. 11470850 10.1093/oxfordjournals.molbev.a003945

[ece310610-bib-0002] Behrens, M. , Brockhoff, A. , Kuhn, C. , Bufe, B. , Winnig, M. , & Meyerhof, W. (2004). The human taste receptor hTAS2R14 responds to a variety of different bitter compounds. Biochemical and Biophysical Research Communications, 319, 479–485. 15178431 10.1016/j.bbrc.2004.05.019

[ece310610-bib-0003] Behrens, M. , Korsching, S. I. , & Meyerhof, W. (2014). Tuning properties of avian and frog bitter taste receptors dynamically fit gene repertoire sizes. Molecular Biology and Evolution, 31, 3216–3227. 25180257 10.1093/molbev/msu254

[ece310610-bib-0004] Borges, R. , Johnson, W. E. , O'Brien, S. J. , Vasconcelos, V. , & Antunes, A. (2012). The role of gene duplication and unconstrained selective pressures in the melanopsin gene family evolution and vertebrate circadian rhythm regulation. PLoS One, 7, e52413. 23285031 10.1371/journal.pone.0052413 PMC3528684

[ece310610-bib-0005] Bufe, B. , Hofmann, T. , Krautwurst, D. , Raguse, J. D. , & Meyerhof, W. (2002). The human TAS2R16 receptor mediates bitter taste in response to beta‐glucopyranosides. Nature Genetics, 32, 397–401. 12379855 10.1038/ng1014

[ece310610-bib-0006] Copley, S. D. (2020). Evolution of new enzymes by gene duplication and divergence. The FEBS Journal, 287, 1262–1283. 32250558 10.1111/febs.15299 PMC9306413

[ece310610-bib-0007] Davis, J. K. , Lowman, J. J. , Thomas, P. J. , ten Hallers, B. F. , Koriabine, M. , Huynh, L. Y. , Maney, D. L. , de Jong, P. J. , Martin, C. L. , & Thomas, J. W. (2010). Evolution of a bitter taste receptor gene cluster in a New World sparrow. Genome Biology and Evolution, 2, 358–370. 20624740 10.1093/gbe/evq027 PMC2942037

[ece310610-bib-0008] Dong, X. , Liang, Q. , Li, J. , & Feng, P. (2021). Positive selection drives the evolution of a primate bitter taste receptor gene. Ecology and Evolution, 11, 5459–5467. 34026020 10.1002/ece3.7440 PMC8131804

[ece310610-bib-0009] Felsenstein, J. (1985). Confidence limits on phylogenies: An approach using the bootstrap. Evolution, 39, 783–791. 28561359 10.1111/j.1558-5646.1985.tb00420.x

[ece310610-bib-0010] Feng, P. , Zheng, J. S. , Rossiter, S. J. , Wang, D. , & Zhao, H. B. (2014). Massive losses of taste receptor genes in toothed and baleen whales. Genome Biology and Evolution, 6, 1254–1265. 24803572 10.1093/gbe/evu095 PMC4079202

[ece310610-bib-0011] Fischer, A. , Gilad, Y. , Man, O. , & Paabo, S. (2005). Evolution of bitter taste receptors in humans and apes. Molecular Biology and Evolution, 22, 432–436. 15496549 10.1093/molbev/msi027

[ece310610-bib-0012] Fleagle, J. G. (1999). Primate adaptation and evolution .

[ece310610-bib-0013] Hayakawa, T. , Suzuki‐Hashido, N. , Matsui, A. , & Go, Y. (2014). Frequent expansions of the bitter taste receptor gene repertoire during evolution of mammals in the Euarchontoglires clade. Molecular Biology and Evolution, 31, 2018–2031. 24758778 10.1093/molbev/msu144

[ece310610-bib-0014] Hu, X. , Wang, G. , Shan, L. , Sun, S. , Hu, Y. , & Wei, F. (2020). TAS2R20 variants confer dietary adaptation to high‐quercitrin bamboo leaves in Qinling giant pandas. Ecology and Evolution, 10, 5913–5921. 32607200 10.1002/ece3.6327 PMC7319149

[ece310610-bib-0015] Huang, Y. F. , & Golding, G. B. (2012). Inferring sequence regions under functional divergence in duplicate genes. Bioinformatics, 28, 176–183. 22121158 10.1093/bioinformatics/btr635

[ece310610-bib-0016] Hughes, J. , & Criscuolo, F. (2008). Evolutionary history of the UCP gene family: Gene duplication and selection. BMC Evolutionary Biology, 8, 306. 18980678 10.1186/1471-2148-8-306 PMC2584656

[ece310610-bib-0017] Imai, H. , Suzuki, N. , Ishimaru, Y. , Sakurai, T. , Yin, L. , Pan, W. , Abe, K. , Misaka, T. , & Hirai, H. (2012). Functional diversity of bitter taste receptor TAS2R16 in primates. Biology Letters, 8, 652–656. 22399783 10.1098/rsbl.2011.1251 PMC3391450

[ece310610-bib-0018] Itoigawa, A. , Fierro, F. , Chaney, M. E. , Lauterbur, M. E. , Hayakawa, T. , Tosi, A. J. , Niv, M. Y. , & Imai, H. (2021). Lowered sensitivity of bitter taste receptors to beta‐glucosides in bamboo lemurs: An instance of parallel and adaptive functional decline in TAS2R16? Proceedings of the Biological Sciences, 288, 20210346. 10.1098/rspb.2021.0346 PMC8059561 33849315

[ece310610-bib-0019] Itoigawa, A. , Hayakawa, T. , Zhou, Y. , Manning, A. D. , Zhang, G. , Grutzner, F. , & Imai, H. (2022). Functional diversity and evolution of bitter taste receptors in egg‐laying mammals. Molecular Biology and Evolution, 39, msac107. 35652727 10.1093/molbev/msac107 PMC9161717

[ece310610-bib-0020] Jiao, H. , Wang, Y. , Zhang, L. , Jiang, P. , & Zhao, H. (2018). Lineage‐specific duplication and adaptive evolution of bitter taste receptor genes in bats. Molecular Ecology, 27, 4475–4488. 30230081 10.1111/mec.14873 PMC8381267

[ece310610-bib-0021] Kitanovic, S. , Orr, T. J. , Spalink, D. , Cocke, G. B. , Schramm, K. , Wilderman, P. R. , Halpert, J. R. , & Dearing, M. D. (2018). Role of cytochrome P450 2B sequence variation and gene copy number in facilitating dietary specialization in mammalian herbivores. Molecular Ecology, 27, 723–736. 29319892 10.1111/mec.14480

[ece310610-bib-0022] Konrad, A. , Teufel, A. I. , Grahnen, J. A. , & Liberles, D. A. (2011). Toward a general model for the evolutionary dynamics of gene duplicates. Genome Biology and Evolution, 3, 1197–1209. 21920903 10.1093/gbe/evr093 PMC3205605

[ece310610-bib-0023] Kuzmin, E. , Taylor, J. S. , & Boone, C. (2022). Retention of duplicated genes in evolution. Trends in Genetics, 38, 59–72. 34294428 10.1016/j.tig.2021.06.016 PMC8678172

[ece310610-bib-0024] Li, D. Y. , & Zhang, J. Z. (2014). Diet shapes the evolution of the vertebrate bitter taste receptor gene repertoire. Molecular Biology and Evolution, 31, 303–309. 24202612 10.1093/molbev/mst219 PMC3907052

[ece310610-bib-0025] Liang, Q. F. , Shu, F. L. , Dong, X. Y. , & Feng, P. (2021). The evolution of a bitter taste receptor gene in primates. Chemical Senses, 46, bjab049. 34864939 10.1093/chemse/bjab049

[ece310610-bib-0026] Liu, Z. , Liu, G. , Hailer, F. , Orozco‐terWengel, P. , Tan, X. , Tian, J. , Yan, Z. , Zhang, B. , & Li, M. (2016). Dietary specialization drives multiple independent losses and gains in the bitter taste gene repertoire of Laurasiatherian mammals. Frontiers in Zoology, 13, 28. 27366197 10.1186/s12983-016-0161-1 PMC4928315

[ece310610-bib-0027] Lossow, K. , Hubner, S. , Roudnitzky, N. , Slack, J. P. , Pollastro, F. , Behrens, M. , & Meyerhof, W. (2016). Comprehensive analysis of mouse bitter taste receptors reveals different molecular receptive ranges for orthologous receptors in mice and humans. Journal of Biological Chemistry, 291, 15358–15377. 27226572 10.1074/jbc.M116.718544 PMC4946946

[ece310610-bib-0028] Magadum, S. , Banerjee, U. , Murugan, P. , Gangapur, D. , & Ravikesavan, R. (2013). Gene duplication as a major force in evolution. Journal of Genetics, 92, 155–161. 23640422 10.1007/s12041-013-0212-8

[ece310610-bib-0029] Meyerhof, W. , Batram, C. , Kuhn, C. , Brockhoff, A. , Chudoba, E. , Bufe, B. , Appendino, G. , & Behrens, M. (2010). The molecular receptive ranges of human TAS2R bitter taste receptors. Chemical Senses, 35, 157–170. 20022913 10.1093/chemse/bjp092

[ece310610-bib-0030] Nei, M. , & Kumar, S. (2000). Molecular evolution and phylogenetics (pp. 87–103). Oxford University Press.

[ece310610-bib-0031] Ohno, S. (1970). Evolution by gene duplication. Springer‐Verlag.

[ece310610-bib-0032] Pegueroles, C. , Laurie, S. , & Alba, M. M. (2013). Accelerated evolution after gene duplication: A time‐dependent process affecting just one copy. Molecular Biology and Evolution, 30, 1830–1842. 23625888 10.1093/molbev/mst083

[ece310610-bib-0033] Perelman, P. , Johnson, W. E. , Roos, C. , Seuánez, H. N. , Horvath, J. E. , Moreira, M. A. , Kessing, B. , Pontius, J. , Roelke, M. , Rumpler, Y. , & Schneider, M. P. (2011). A molecular phylogeny of living primates. PLoS Genetics, 7, e1001342. 21436896 10.1371/journal.pgen.1001342 PMC3060065

[ece310610-bib-0034] Sainz, E. , Cavenagh, M. M. , Gutierrez, J. , Battey, J. F. , Northup, J. K. , & Sullivan, S. L. (2007). Functional characterization of human bitter taste receptors. Biochemical Journal, 403, 537–543. 17253962 10.1042/BJ20061744 PMC1876383

[ece310610-bib-0035] Saitou, N. , & Nei, M. (1987). The neighbor‐joining method: A new method for reconstructing phylogenetic trees. Molecular Biology and Evolution, 4, 406–425. 3447015 10.1093/oxfordjournals.molbev.a040454

[ece310610-bib-0036] Shang, S. , Zhang, H. X. , Wu, X. Y. , Chen, J. , Zhong, H. M. , Wei, Q. G. , Zhao, C. , Yan, J. K. , Chen, Y. , Tang, X. X. , et al. (2017). The repertoire of bitter taste receptor genes in Ovalentaria fish. Environmental Biology of Fishes, 100, 1489–1496.

[ece310610-bib-0037] Shi, P. , Zhang, J. , Yang, H. , & Zhang, Y. P. (2003). Adaptive diversification of bitter taste receptor genes in mammalian evolution. Molecular Biology and Evolution, 20, 805–814. 12679530 10.1093/molbev/msg083

[ece310610-bib-0038] Song, Y. , Gao, J. , Yang, F. , Kua, C. S. , Liu, J. , & Cannon, C. H. (2013). Molecular evolutionary analysis of the alfin‐like protein family in *Arabidopsis lyrata*, *Arabidopsis thaliana*, and *Thellungiella halophila* . PLoS One, 8, e66838. 23840867 10.1371/journal.pone.0066838 PMC3698079

[ece310610-bib-0039] Strain, E. , & Muse, S. V. (2005). Positively selected sites in the *Arabidopsis* receptor‐like kinase gene family. Journal of Molecular Evolution, 61, 325–332. 16044247 10.1007/s00239-004-0308-0

[ece310610-bib-0040] Tamura, K. , Stecher, G. , Peterson, D. , Filipski, A. , & Kumar, S. (2013). MEGA6: Molecular evolutionary genetics analysis version 6.0. Molecular Biology and Evolution, 30, 2725–2729. 24132122 10.1093/molbev/mst197 PMC3840312

[ece310610-bib-0041] Wacholder, A. , & Carvunis, A. R. (2021). New genes from borrowed parts. Science, 371, 779–780. 33602841 10.1126/science.abf8493

[ece310610-bib-0042] Wang, K. , & Zhao, H. B. (2015). Birds generally carry a small repertoire of bitter taste receptor genes. Genome Biology and Evolution, 7, 2705–2715. 26342138 10.1093/gbe/evv180 PMC4607536

[ece310610-bib-0043] Wang, X. X. , Thomas, S. D. , & Zhang, J. Z. (2004). Relaxation of selective constraint and loss of function in the evolution of human bitter taste receptor genes. Human Molecular Genetics, 13, 2671–2678. 15367488 10.1093/hmg/ddh289

[ece310610-bib-0044] Wang, Y. , Jiao, H. , Jiang, P. , & Zhao, H. (2019). Functional divergence of bitter taste receptors in a nectar‐feeding bird. Biology Letters, 15, 20190461. 31551065 10.1098/rsbl.2019.0461 PMC6769140

[ece310610-bib-0045] Wooding, S. (2011). Signatures of natural selection in a primate bitter taste receptor. Journal of Molecular Evolution, 73, 257–265. 22218679 10.1007/s00239-011-9481-0

[ece310610-bib-0046] Wooding, S. , Gunn, H. , Ramos, P. , Thalmann, S. , Xing, C. , & Meyerhof, W. (2010). Genetics and bitter taste responses to goitrin, a plant toxin found in vegetables. Chemical Senses, 35, 685–692. 20551074 10.1093/chemse/bjq061

[ece310610-bib-0047] Yang, Z. (2000). Complexity of the simplest phylogenetic estimation problem. Proceedings of the Biological Sciences, 267, 109–116. 10.1098/rspb.2000.0974 PMC1690513 10687814

[ece310610-bib-0048] Yang, Z. , & Nielsen, R. (2002). Codon‐substitution models for detecting molecular adaptation at individual sites along specific lineages. Molecular Biology and Evolution, 19, 908–917. 12032247 10.1093/oxfordjournals.molbev.a004148

[ece310610-bib-0049] Yang, Z. , Wong, W. S. , & Nielsen, R. (2005). Bayes empirical bayes inference of amino acid sites under positive selection. Molecular Biology and Evolution, 22, 1107–1118. 15689528 10.1093/molbev/msi097

[ece310610-bib-0050] Zhang, J. (2003). Evolution by gene duplication: An update. Trends in Ecology & Evolution, 18, 292–298.

[ece310610-bib-0051] Zhang, J. , Nielsen, R. , & Yang, Z. (2005). Evaluation of an improved branch‐site likelihood method for detecting positive selection at the molecular level. Molecular Biology and Evolution, 22, 2472–2479. 16107592 10.1093/molbev/msi237

[ece310610-bib-0052] Zhao, S. , Zheng, P. , Dong, S. , Zhan, X. , Wu, Q. , Guo, X. , Hu, Y. , He, W. , Zhang, S. , Fan, W. , Zhu, L. , Li, D. , Zhang, X. , Chen, Q. , Zhang, H. , Zhang, Z. , Jin, X. , Zhang, J. , Yang, H. , … Wei, F. (2013). Whole‐genome sequencing of giant pandas provides insights into demographic history and local adaptation. Nature Genetics, 45, 67–71. 23242367 10.1038/ng.2494

[ece310610-bib-0053] Zhong, H. , Shang, S. , Wu, X. , Chen, J. , Zhu, W. , Yan, J. , Li, H. , & Zhang, H. (2017). Genomic evidence of bitter taste in snakes and phylogenetic analysis of bitter taste receptor genes in reptiles. PeerJ, 5, e3708. 28828281 10.7717/peerj.3708 PMC5564386

[ece310610-bib-0054] Zhong, H. M. , Huang, J. , Shang, S. , & Yuan, B. D. (2021). Evolutionary insights into umami, sweet, and bitter taste receptors in amphibians. Ecology and Evolution, 11, 18011–18025. 35003653 10.1002/ece3.8398 PMC8717283

