# Supplementary material for: The evolution of bitter taste receptor gene in primates: Gene duplication and selection
Source: Ecol Evol. 2023 Oct 13;13(10):e10610. doi: 10.1002/ece3.10610 (PMC10571502; doi:10.1002/ece3.10610)
Supplement: Supplementary file 2 — Table S1. [file ECE3-13-e10610-s001.docx]

**Appendix table S1 *T2R* gene used in this study.**

| Gene name | Genes of different species | Prefix: Species name |
| --- | --- | --- |
| *T2R1* | Mamu_971_670, Mane_137_836, Mafa_670_369, Paan_523_422, Thge_779_678, Male_634_333, Ceat_450_149, Chsa_286_185, Pite_051_950, Trfr_168_067, Pyne_161_060, Rhbi_760_659, Rhro_396_095, Nala_066_965, Nole_726_425, Poab_074_973, Gogo_810_709, Hosa_hT2R1_NM_019599, Patr_869_768, Papa_630_329, Caja_072_771, Aona_464_363, Sabo_275_174, Ceca_608_307, Tasy_952_651, Dama_232_131, Prco_899_592, Prsi_998_885, Eufl_640_527, Euma_499_386, Mimu_386_085, Otga_984_063 | Mamu:  *Macaca mulatta*  Mafu:  *Macaca fuscata*  Mafa:  *Macaca fascicularis*  Mane:  *Macaca nemestrina*  Poab:  *Pongo abelii*  Thge:  *Theropithecus gelada*  Male:  *Mandrillus leucophaeus*  Ceat:  *Cercocebus atys*  Chsa:  *Chlorocebus sabaeus*  Pite:  *Piliocolobus tephrosceles*  Coan:  *Colobus angolensis*  Trfr:  *Trachypithecus francoisi*  Pyne:  *Pygathrix nemaeus*  Nala:  *Nasalis larvatus*  Rhbi:  *Rhinopithecus bieti*  Rhro:  *Rhinopithecus roxellana*  Nole:  *Nomascus leucogenys*  Paan:  *Papio anubis*  Gogo:  *Gorilla gorilla*  Hosa:  *Homo sapiens*  Patr:  *Pan troglodytes*  Papa:  *Pan paniscus*  Caja:  *Callithrix jacchus*  Aona:  *Aotus nancymaae*  Sabo:  *Saimiri boliviensis*  Ceca:  *Cebus capucinus*  Tasy:  *Tarsius syrichta*  Otga:  *Otolemur garnettii*  Dama:  *Daubentonia madagascariensis*  Prco:  *Propithecus coquereli*  Mimu:  *Microcebus murinus*  Prsi:  *Prolemur simus*  Eufl:  *Eulemur flavifrons*  Euma:  *Eulemur macaco* |
| *T2R2* | Mamu_181_895, Mafa_929_643, Mafu_572_486, Mane_373_081, Thge_409_323, Chsa_923_837, Male_675_389, Ceat_378_509, Pite_955_869, Coan_941_855, Trfr_332_246, Pyne_537_451, Nala_659_573, Rhbi_003_917, Rhro_448_162, Nole_971_682, Gogo_744_655, Patr_279_190, Papa_721_432, Poab_067_978, Tasy_996_704, Otga_777_886, Dama_447_364, Mimu_118_823, Prsi_506_417, Eufl_896_819, Euma_610_533, Caja_780_485, Aona_112_149, Ceca_062_767, Sabo_446_483 |  |
| *T2R3* | Mamu_181_931, Mafu_036_983, Mafa_441_191, Mane_678_428, Male_051_801, Ceat_853_603, Paan_348_298, Thge_555_505, Chsa_303_253, Pite_457_407, Coan_444_394, Trfr_318_529, Nala_831_781, Pyne_730_680, Rhbi_671_621, Rhro_192_942, Nole_151_901, Poab_116_066, Gogo_567_553, Hosa_hT2R3_NM_016943, Patr_794_867, Papa_251_947, Caja_864_611, Sabo_408_358, Ceca_446_196, Aona_091_041, Tasy_474_209, Otga_833_783, Dama_853_103, Prco_274_024, Prsi_730_680, Eufl_562_512, Euma_302_252, Mimu_709_459 |  |
| *T2R4* | Mamu_331_030, Mafu_188_087, Mafa_580_279, Mane_595_294, Paan_576_475, Thge_344_243, Male_289_988, Ceat_972_671, Chsa_134_033, Pite_554_453, Trfr_824_723, Pyne_543_442, Rhbi_483_382, Rhro_428_127, Nala_174_073, Coan_247_146, Caja_828_527, Aona_030_929, Sabo_009_908, Ceca_558_257, Tasy_902_601, Otga_713_606, Dama_571_464, Prco_912_605, Mimu_057_750, Prsi_875_768, Eufl_949_842, Euma_689_582, Nole_262_961, Gogo_870_769, Hosa_hT2R4_NM_016944, Patr_009_908, Papa_411_110, Poab_474_373 |  |
| *T2R5* | Mamu_386_085, Mafa_647_346, Mafu_330_229, Mane_729_428, Ceat_060_759, Male_844_543, Paan_156_055, Thge_718_617_2, Chsa_732_631, Pite_096_995, Coan_872_771, Trfr_426_325, Rhbi_985_884, Rhro_768_467, Pyne_908_807, Nole_038_737, Poab_361_260, Gogo_722_621, Patr_813_712, Papa_331_030, Hosa_hT2R5_NM_018980, Caja_220_919, Aona_912_811, Sabo_857_756, Ceca_088_787, Tasy_283_982, Prco_973_666 |  |
| *T2R7* | Mamu_490_246, Mafa_245_980, Mafu_467_444, Mane_505_261, Paan_896_849, Male_816_572, Ceat_158_914, Thge_749_705, Chsa_391_347, Pite_633_589, Trfr_526_482, Pyne_917_023, Nala_199_155, Rhbi_240_196, Rhro_702_458, Nole_444_200, Poab_531_487, Gogo_118_074, Hosa_hT2R7_NM_023919, Patr_736_815, Papa_493_246, Caja_096_852, Caja_706_345, Aona_160_116, Sabo_190_128, Ceca_444_209, Otga_038_982, Dama_429_385, Prco_480_236, Mimu_045_801, Prsi_367_305, Eufl_306_262, Euma_033_989 |  |
| *T2R8* | Mamu_073_829, Mane_257_013, Mafu_462_418, Mafa_646_402, Thge_229_185, Male_371_127, Ceat_011_767, Paan_343_272, Pite_202_131, Trfr_123_052, Pyne_449_378, Nala_651_580, Rhbi_672_601, Rhro_289_018, Coan_565_494, Chsa_962_891, Nole_543_299, Gogo_532_488, Hosa_hT2R8_NM_023918, Patr_212_168, Papa_932_661, Poab_446_375, Caja_800_529, Caja_320_049, Aona_444_400, Sabo_901_830, Ceca_655_411, Tasy_780_509, Dama_881_810, Prco_829_558, Prsi_835_764 |  |
| *T2R9* | Mamu_983_688, Mafa_760_495, Mafu_576_511, Mane_368_103, Male_487_222, Ceat_122_857, Chsa_045_980, Paan_247_182, Thge_138_073, Coan_449_420, Nala_732_670, Nole_448_186, Poab_531_469, Hosa_hT2R9_NM_023917, Patr_321_259, Aona_490_422, Sabo_841_773, Ceca_704_385 |  |
| *T2R10* | Mamu_721_444, Mafa_053_776, Mafu_863_786, Mane_650_373, Male_772_495, Ceat_183_885, Chsa_627_559, Paan_413_336, Thge_176_099, Pite_805_728, Trfr_273_196, Pyne_023_078, Rhbi_749_672, Rhro_186_909, Nala_832_752, Coan_058_981, Nole_490_213, Poab_658_638, Gogo_817_740, Hosa_hT2R10_NM_023921, Patr_422_345, Papa_055_778, Caja_624_347, Sabo_135_058, Ceca_141_870, Aona_133_056, Tasy_502_228, Otga_548_561, Otga_767_792, Otga_012_932, Otga_727_641, Otga_707_633, Mimu_901_627, Eufl_740_711, Euma_601_527, Tasy_128_860 |  |
| *T2R12* | Otga_608_654, Mimu_304_042, Eufl_055_993, Euma_055_993, Dama_720_688, Prco_925_663, Mimu_121_838, Prsi_798_739, Eufl_408_225, Euma_370_087 |  |
| *T2R13* | Mamu_682_393, Mafu_649_632, Mane_781_492, Mafa_248_959, Paan_048_959, Thge_241_152, Male_518_229, Ceat_488_199, Chsa_842_753, Pite_225_136, Coan_786_694, Trfr_188_099, Rhbi_254_165, Rhro_036_747, Nala_886_797, Nole_150_861, Poab_505_416, Gogo_271_182, Hosa_hT2R13_NM_023920, Patr_080_991, Papa_723_434, Caja_925_636, Caja_381_092, Sabo_673_590, Aona_600_592, Aona_171_082, Ceca_705_416, Sabo_724_635, Tasy_441_152, Otga_079_134, Otga_942_997, Dama_138_049, Dama_406_317, Prco_281_992, Mimu_791_502, Mimu_706_417, Prsi_961_872, Prsi_598_509, Eufl_752_663, Euma_751_662 |  |
| *T2R14* | Mamu_033_798, Mafu_283_242, Mafa_411_176, Mane_352_111, Ceat_096_855, Paan_892_851, Paan_086_045, Thge_876_835, Male_780_539, Chsa_148_107, Pite_281_240, Coan_840_922, Trfr_329_291, Pyne_852_811, Nala_550_509, Rhro_197_956, Nole_368_124, Poab_776_732, Gogo_192_145, Hosa_hT2R14_NM_023922, Patr_863_816, Papa_509_262, Aona_378_337, Ceca_703_462, Tasy_742_492, Tasy_762_521, Otga_146_105, Dama_176_135, Mimu_004_763, Eufl_354_313, Euma_349_308 |  |
| *T2R16* | Mamu_049_724, Mafu_012_887, Mafa_252_927, Mane_593_268, Paan_555_430, Thge_646_515, Male_360_035, Ceat_145_820, Chsa_058_002, Pite_933_808, Nala_956_831, Trfr_512_387, Pyne_857_732, Rhbi_613_488, Rhro_564_239, Coan_497_372, Nole_546_221, Poab_996_940, Gogo_574_449, Hosa_hT2R16_NM_016945, Papa_999_674, Patr_859_734, Caja_679_357, Aona_275_153, Ceca_458_136, Tasy_811_495, Otga_545_624, Dama_792_697, Prco_424_129, Mimu_903_608, Prsi_987_892, Eufl_449_354, Euma_911_816 |  |
| *T2R38* | Mamu_963_764, Mafu_594_595, Mafa_914_715, Mane_942_743, Chsa_518_519, Thge_120_121, Male_618_419, Ceat_863_664, Coan_956_957, Trfr_683_821, Pyne_766_767, Nala_T2R38, Rhbi_812_813, Rhro_640_441, Paan_618_619, Nole_764_565, Poab_564_565, Gogo_571_572, Hosa_hT2R38_NM_176817, Patr_253_329, Papa_627_428, Caja_201_002, Sabo_972_973, Aona_624_625, Tasy_155_953, Otga_122_123, Prco_209_010, Prsi_797_798, Eufl_032_870, Euma_420_415, Mimu_376_177 |  |
| *T2R39* | Mamu_315_131, Mane_534_350, Mafa_602_418, Paan_601_617, Thge_520_536, Male_963_779, Ceat_855_671, Chsa_368_384, Pite_608_630, Coan_907_923, Nole_082_898, Gogo_174_190, Hosa_hT2R39_NM_176881, Patr_016_032, Papa_004_820, Caja_766_579, Aona_878_897, Sabo_226_305, Ceca_995_808, Mimu_929_637, Prsi_499_231, Eufl_905_873, Euma_887_855 |  |
| *T2R40* | Mamu_431_202, Mafu_774_745, Mafa_635_406, Mane_896_667, Paan_197_168, Thge_974_945, Male_491_262, Ceat_887_658, Chsa_400_371, Pite_119_090, Trfr_027_998, Pyne_474_445, Nala_088_059, Rhbi_268_239, Rhro_805_576, Coan_583_554, Nole_883_654, Poab_250_221, Gogo_841_812, Hosa_hT2R40_NM_176882, Patr_608_579, Papa_619_381, Caja_628_399, Aona_117_088, Sabo_413_384, Ceca_190_961, Tasy_722_490, Otga_362_330, Dama_094_365, Prco_993_764, Prsi_967_938, Eufl_474_445, Euma_209_174, Mimu_671_442 |  |
| *T2R41* | Mamu_668_391, Mane_450_173, Mafu_153_076, Mafa_748_471, Paan_032_955, Thge_490_413, Male_440_163, Ceat_936_659, Chsa_760_683, Pite_905_828, Trfr_788_711, Pyne_499_422, Rhbi_404_327, Rhro_990_713, Nala_264_187, Nole_284_007, Gogo_989_912, Hosa_hT2R41_NM_176883, Patr_679_602, Papa_255_978, Poab_841_764, Caja_370_093, Ceca_742_465, Sabo_484_464, Aona_368_291, Tasy_659_385, Tasy_594_320, Otga_004_116, Otga_603_712, Dama_447_373, Prco_772_498, Prco_739_465, Mimu_466_192, Prsi_837_763, Eufl_764_741, Euma_559_485 |  |
| *T2R42* | Mamu_525_269, Mafu_838_902, Mafa_108_852, Mane_293_037, Male_087_831, Ceat_995_739, Paan_058_002, Thge_264_208, Chsa_050_994, Coan_058_002, Trfr_357_301, Nala_523_467, Rhbi_971_915, Rhro_957_701, Poab_467_411, Gogo_845_978, Hosa_hT2R42_NM_181429, Patr_055_999, Papa_063_807, Caja_005_749, Sabo_082_026, Ceca_326_070, Tasy_901_642, Otga_126_067, Dama_400_341, Prco_078_816, Prco_588_293, Prco_683_388, Prco_917_649, Mimu_028_766, Mimu_367_084, Mimu_690_422, Mimu_960_692, Mimu_769_510, Prsi_734_666, Prsi_422_363, Eufl_997_938, Euma_979_920, Mimu_376_105, Prsi_666_586, Euma_580_500, Prsi_936_871 |  |
| *T2R60* | Mamu_485_241, Mafu_173_129, Mafa_619_375, Mane_811_567, Chsa_191_147, Paan_417_373, Thge_019_975, Male_971_727, Coan_837_793, Pyne_869_825, Nole_996_752, Poab_357_313, Gogo_262_218, Hosa_hT2R60_NM_177437, Patr_131_087, Papa_810_566, Sabo_337_293, Otga_947_080, Dama_986_194, Prco_647_403, Mimu_394_144, Prsi_792_835, Eufl_756_799, Euma_756_799 |  |
| *T2R62* | Mamu_048_705, Mafu_729_667, Mafa_188_902, Mane_321_029, Male_550_264, Ceat_210_924, Paan_904_815, Thge_579_490, Chsa_762_676, Pite_350_264, Coan_409_323, Trfr_674_588, Nala_938_852, Rhbi_530_444, Rhro_404_118, Pyne_318_232, Nole_575_286, Poab_897_934, Gogo_495_472, Patr_648_685, Papa_392_103, Caja_256_970, Aona_566_480, Sabo_739_716, Ceca_583_297, Tasy_893_559, Otga_026_132, Dama_454_362, Prco_135_870, Mimu_901_639, Eufl_656_609, Euma_656_609 |  |
| *T2R30*/*T2R46*  (clade1_a1-b2) | Pite_730_656, Pite_621_550, Nala_255_181, Rhbi_892_818, Rhro_525_251, Trfr_851_777, Paan_723_649, Chsa_584_510, Thge_692_618, Male_840_566, Ceat_094_820, Mafa_480_203, Mane_978_704, Mamu_136_862, Mafu_609_535,  Mamu_814_543, Mafu_813_703, Paan_757_725, Male_528_257, Ceat_399_128, Ceat_619_348, Chsa_392_321, Nala_467_396, Rhbi_8_033, Coan_296_228, Chsa_628_557, Paan_489_418,  Nole_151_859, Poab_193_191, Gogo_126_130, Patr_172_203, Papa_064_772, Hosa_hT2R30_NM_001097643,  Gogo_203_132, Patr_798_727, Papa_257_986, Hosa_hT2R46_NM_176887 |  |
| *T2R31*/*T2R43*  (clade1_c-c2) | Poab_295_224, Gogo_408_337, Hosa_hT2R31_NM_176885, Papa_557_286, Hosa_hT2R43_NM_176884, Patr_771_700,  Mamu_917_646, Mafu_498_427, Mafa_899_628, Mane_232_961, Male_194_923, Ceat_097_826_1, Paan_287_216, Chsa_830_759, Thge_190_119, Trfr_748_677, Pyne_088_017, Nala_276_205, Rhro_383_112, Pite_016_945, Coan_678_607,  Mamu_376_102, Mafa_514_240, Mane_523_249, Mafu_718_644, Paan_050_976, Thge_614_540, Male_093_819, Chsa_142_068, Pite_332_258, Coan_275_201, Trfr_609_535, Pyne_327_253, Nala_071_997, Rhbi_311_237, Rhro680_406 |  |
| *T2R45*  (clade1_d1-d3) | Mamu_648_377, Mafa_628_357, Mane_595_324, Paan_185_183, Thge_629_651, Male_183_912, Ceat_097_826_2, Chsa_585_514, Pite_930_859, Coan_178_107, Trfr_187_116, Pyne_530_459, Nala_773_702, Rhro_493_222,  Mamu_607_336, Mafu_959_888, Mafa_757_486, Thge_868_797, Ceat_250_979, Mane_558_287, Paan_467_396, Male_200_929, Pite_193_122, Trfr_387_316, Nala_857_786, Pyne_500_429, Nala_975_904, Rhro_513_242, Coan_412_341, Chsa_724_653,  Mamu_740_469, Mafu_253_182, Mafa_409_138, Mafu_622_551, Paan_021_950, Thge_684_613, Ceat_972_701, Male_453_182, Mane_934_663, Ceat_293_022, Chsa_468_790, Male_879_608, Ceat_501_230, Trfr_341_270, Rhbi_599_528, Rhro_042_771 |  |
| *T2R50*  (clade1_e1-e2) | Paan_621_853, Paan_069_325, Thge_830_906, Male_879_581, Ceat_531_227, Coan_054_130, Poab_618_538, Sabo_768_670, Ceca_180_882,  Mamu_356_085, Mafu_704_633, Male_322_021, Mafa_534_233, Mane_583_312, Ceat_723_446, Chsa_051_950, Paan_858_787, Thge_718_617_1, Pite_672_598, Trfr_668_597, Nala_046_945, Rhbi_884_783, Rhro_891_590, Coan_328_227, Nole_250_949, Poab_571_500, Gogo_898_827, Hosa_hT2R50_NM_176890, Patr_435_364, Papa_053_782, Caja_389_118, Sabo_388_287, Ceca_825_554, Aona_019_948 |  |
| *T2R19/T2R20*  (clade1_f1-f2) | Mamu_025_754, Male_739_438, Ceat_174_903, Thge_621_520, Chsa_709_608, Mafa_353_052, Mane_786_515, Patr_775_704, Poab_919_848, Gogo_658_557, Patr_184_113, Papa_767_496, Hosa_hT2R19_NM_176888,  Mamu_450_179, Mafa_603_332, Mafu_789_718, Mane_415_144, Paan_899_828, Thge_679_608, Male_659_388, Ceat_599_328, Chsa_101_030, Coan_291_310, Trfr_881_810, Nala_229_158, Rhro_234_963, Nole_230_959, Gogo_908_837, Hosa_hT2R20_NM_176889, Patr_419_348, Papa_036_765, Poab_569_498, Caja_149_875, Sabo_416_342, Ceca_817_543 |  |

**Appendix table S2 Positively selected sites detected by site-model in clade with one-to-one orthologous genes. All p-values are smaller than 0.05 except in *T2R38* cluster (M1a vs M2a, M8 vs M8a), all the comparisons of *T2R39*, and *T2R40* (M1a vs M2a).**

| Gene cluster/clade | Positively selected sites (BEB)^a^ |
| --- | --- |
| *T2R1* | M2a:  77 M, 80 A, 83 A, 85 L, 86 L, 150 F, 167 T, 168 L, 241 V, 254 I |
|  | M8:  9 Y, 77 M, 80 A, 83 A, 85 L, 86 L, 148 A, 149 G, 150 F, 154 Y, 158 K, 167 T, 168 L, 170 I, 202 R, 241 V, 250 I, 254 I |
| *T2R2* | M2a:  none |
|  | M8:  79 K, 170 K |
| *T2R3* | M2a:  74 T, 145 A, 271 T |
|  | M8:  74 T, 145 A, 271 T |
| *T2R4* | M2a:  3 R, 70 V, 74 T, 75 E, 85 V, 264 L |
|  | M8:  3 R, 70 V, 74 T, 75 E, 82 A, 85 V, 162 T, 170 E, 206 Q, 220 T, 264 L |
| *T2R5* | M2a:  142 Q, 146 T, 149 H, 171 Q |
|  | M8:  75 R, 142 Q, 146 T, 147 F, 149 H, 171 Q, 235 M |
| *T2R7* | M2a:  209 R |
|  | M8:  209 R |
| *T2R9* | M2a:  none |
|  | M8:  73 G, 78 S, 79 V, 86 V, 145 V |
| *T2R16* | M2a:  61 A, 69 S, 178 T |
|  | M8:  61 A, 69 S, 175 Q, 178 T, 243 I |
|  |  |
| *T2R38* | M2a:  none |
|  | M8:  74 S |
| *T2R39* | M2a:  none |
|  | M8:  none |
| *T2R40* | M2a:  none |
|  | M8:  none |
| *T2R60* | M2a:  none |
|  | M8:  2 K, 220 R |
| *T2R62* | M2a:  75 P, 136 E, 149 Q, 166 V, 289 Q, 295 D, 296 A |
|  | M8:  74 V, 75 P, 77 M, 136 E, 142 T, 148 G, 149 Q, 166 V, 168 A, 170 F, 248 I, 287 W, 288 P, 289 Q, 291 Q, 292 C, 294 C, 295 D, 296 A, 297 G |
| clade1_e1-e2 (*T2R50*) | M2a:  none |
|  | M8:  121 T |

**Appendix table S3 Positively selected sites detected by site-model and the improved branch-site model in clade with one-to-more orthologous genes. All p-values of site-model are smaller than 0.05.**

| Gene cluster/clade | Positively selected sites (BEB)^a^ |
| --- | --- |
| *T2R8* | M2a:  124 W, 154 C |
|  | M8:  84 V, 86 F, 124 W, 154 C |
|  | Branch-site:  none (p= 0.63) |
| *T2R10* | M2a:  **68 Q**, 140 S, 146 Y, 148 A, 152 Y, 161 Y, 243 C |
|  | M8:  **68 Q**, 81 I, 91 G, 140 S, 146 Y, 148 A, 152 Y, 161 Y, 243 C |
|  | Branch-site:  **68 Q**, 81 I, 171 L (**p= 2.69E-07**) |
| *T2R12* | M2a:  none |
|  | M8:  175 N |
|  | Branch-site:  none |
| *T2R13* | M2a:  69 A, 72 Y, 78 S, 81 G, 85 M, **158 R**, 172 E, 176 V, **179 K**, 205 Q, 255 L, 258 N, 260 V, **270 V**, 293 L |
|  | M8:  69 A, 72 Y, 78 S, 81 G, 85 M, **158 R**, 159 Y, 172 E, 176 V, **179 K**, 205 Q, 254 E, 255 L, 258 N, 260 V, **270 V**, 293 L |
|  | Branch-site:  **158 R**, **179 K**, **270 V**(**p=1.74E-08**) |
| *T2R14* | M2a:  77 A, 177 S, 202 I, 209 R, 218 I, 244 S, 249 I, 253 T, 256 R |
|  | M8:  70 V, 77 A, 171 N, 177 S, 178 L, 187 I, 188 F, 202 I, 209 R, 218 I, 244 S, 249 I, 253 T, 256 R, 266 Q |
|  | Branch-site:  None |
| *T2R41* | M2a:  73 K, 79 G, 81 G, 255 A, 263 D |
|  | M8:  14 S, 73 K, 79 G, 81 G, 86 H, 158 E, 212 Q, 255 A, 256 K, 263 D, 291 R |
|  | Branch-site:  none |
| *T2R42* | M2a:  **65 D**, 69 V, 72 A, 79 Y, 82 G, 84 T, 86 I, **90 H**, 145 L, 147 L, 152 D, **167 D**, 170 K, 247 M, 248 L, 249 W, 251 N, 253 C, **254 I**, **258 M**, 259 L, 263 A |
|  | M8:  19 I, **65 D**, 69 V, 72 A, 79 Y, 82 G, 84 T, 86 I, **90 H**, 91 M, 92 T, 145 L, 147 L, 152 D, **167 D**, 170 K, 238 L, 242 N, 247 M, 248 L, 249 W, 250 N, 251 N, 252 K, 253 C, **254 I**, **258 M**, 259 L, 260 A, 261 L, 263 A, 272 G |
|  | Branch-site:  46 F, **65 D**, **90 H**, 95 L, **167 D**, 225 M, **254 I**, **258 M** (**p= 1.54E-14**) |
| clade1_a1-b2 (*T2R30*\*T2R46*) | M2a:  **89 A**, **99 A**, **210 L**, **243 S**, 252 G, **255 K**, **262 C**, **266 I**, **291 V** |
|  | M8:  73P, 84A, **89A**, **99A**, 162V, 176M, 179T, **210L**, **243S**, 252G, **255K**, **262C**, **266I**, **291V** |
|  | Branch-site:  **89 A**, **99 A**, **210 L**, **243 S**, **255 K**, **262 C**, **266 I**, **291 V** (**p=5.13E-21**) |
| clade1_c-c2 (*T2R31*\*T2R43*) | M2a:  172 V, **295 Q** |
|  | M8:  33 I, 68 S, 84 A, 154 R, 172 V, **295 Q** |
|  | Branch-site:  **295 Q** (**p=7.39E-04**) |
| clade1_d1-d3 (*T2R45*) | M2a:  **151 Q**, **154 W**, **183 A**, 268 G, **296 Q** |
|  | M8:  11 I, **151 Q**, **154 W**, **183 A**, 268 G, 279 L, 295 W, **296 Q** |
|  | Branch-site:  **151 Q**, **154 W**, **183 A**, **296 Q** (**p= 5.55E-10**) |
| clade1_f1-f2 (*T2R19*/*T2R20*) | M2a:  75 Y, **80 R**, 81 I, 146 I, 172 H, 246 T, **251 L**, 253 T, 256 S, 265 V |
|  | M8:  2 C, 75 Y, **80 R**, 81 I, 146 I, 148 M, 172 H, 178 V, 246 T, **251 L**, 253 T, 256 S, 265 V |
|  | Branch-site:  **80 R**, **251 L** (**p=** **4.10E-05**) |
