# Supplementary material for: The evolution of bitter taste receptor gene in primates: Gene duplication and selection
Source: Ecol Evol. 2023 Oct 13;13(10):e10610. doi: 10.1002/ece3.10610 (PMC10571502; doi:10.1002/ece3.10610)

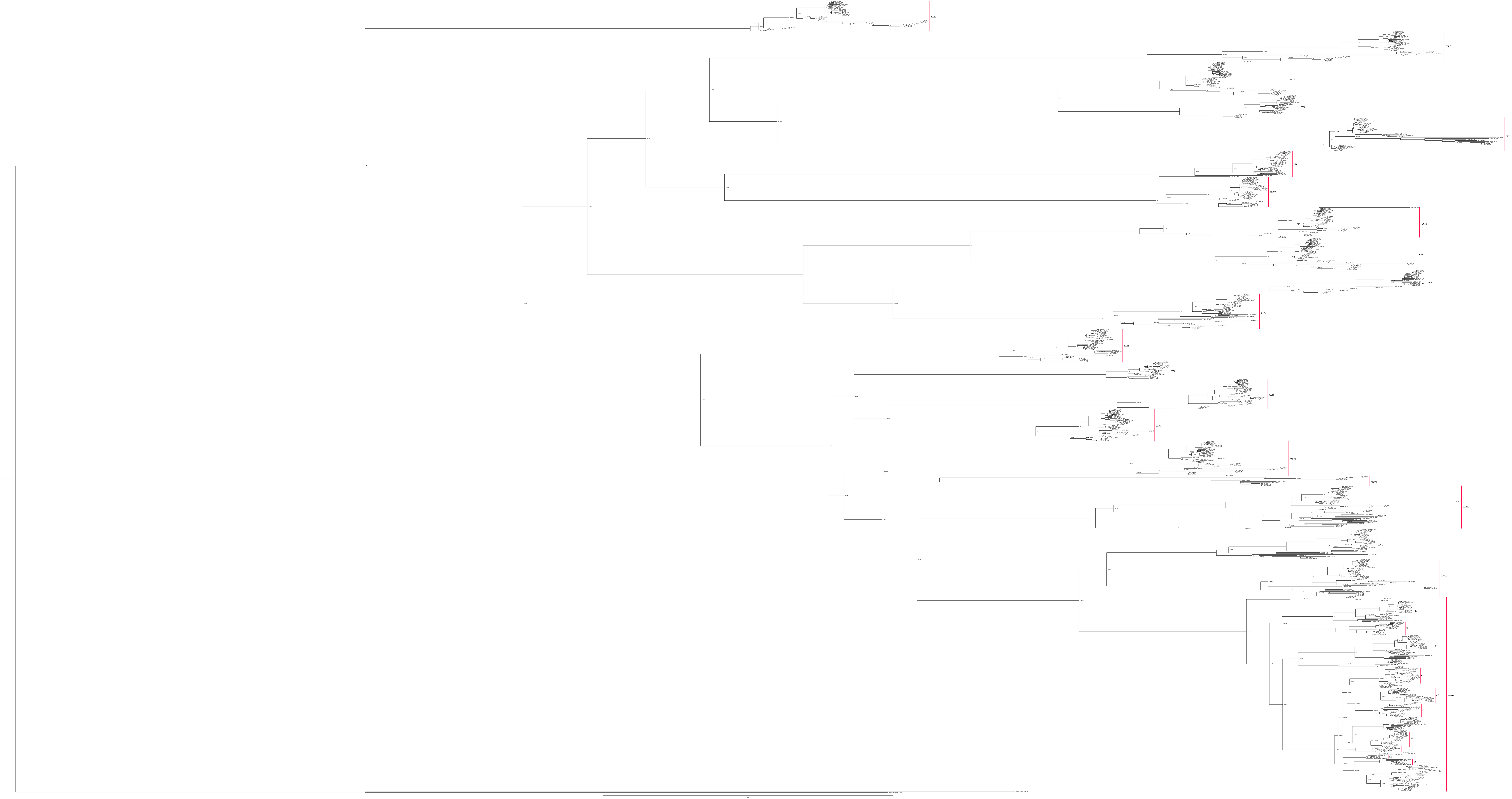

Supplement: Supplementary file 1 — Figure S1. [file ECE3-13-e10610-s002.pdf]
